# Quantitative Assessment of the Intracranial Vasculature of Infants and Adults Using iCafe (Intracranial Artery Feature Extraction)

**DOI:** 10.3389/fneur.2021.668298

**Published:** 2021-05-28

**Authors:** Li Chen, Dennis W. W. Shaw, Stephen R. Dager, Neva M. Corrigan, Baocheng Chu, Natalia M. Kleinhans, Patricia K. Kuhl, Jenq-Neng Hwang, Chun Yuan

**Affiliations:** University of Washington, Seattle, WA, United States

**Keywords:** magnetic resonance angiography, vascular change, feature extraction, brain development, pediatric vascular disease

## Abstract

Comprehensive quantification of intracranial artery features may help to assess and understand regional variations of blood supply during early brain development and aging. We analyzed vasculature features of 27 healthy infants during natural sleep, 13 infants at 7-months (7.3 ± 1.0 month), and 14 infants at 12-months (11.7 ± 0.4 month), and 13 older healthy, awake adults (62.8 ± 8.7 years) to investigate age-related vascular differences as a preliminary study of vascular changes associated with brain development. 3D time-of-flight (TOF) magnetic resonance angiography (MRA) acquisitions were processed in iCafe, a technique to quantify arterial features (http://icafe.clatfd.cn), to characterize intracranial vasculature. Overall, adult subjects were found to have increased ACA length, tortuosity, and vasculature density compared to both 7-month-old and 12-month-old infants, as well as MCA length compared to 7-month-old infants. No brain laterality differences were observed for any vascular measures in either infant or adult age groups. Reduced skull and brain sharpness, indicative of increased head motion and brain/vascular pulsation, respectively, were observed in infants but not correlated with length, tortuosity, or vasculature density measures. Quantitative analysis of TOF MRA using iCafe may provide an objective approach for systematic study of infant brain vascular development and for clinical assessment of adult and pediatric brain vascular diseases.

## Introduction

Three-dimensional time-of-flight (TOF) magnetic resonance angiography (MRA) is a reliable technique for quantitative vascular analysis (1) that also can be obtained without the need for contrast agents or radiation exposure, which is especially desirable for pediatric studies. Quantitative intracranial artery analysis has potential to systematically investigate vascular characteristics and distribution during early infant brain development.

Intracranial artery morphometric analysis has previously primarily focused on healthy adults with relatively good image quality. Bullitt et al. analyzed the effects of aging on intracranial vasculature in 100 healthy volunteers aged 18–74 and found a lower number of MRA-discernible vessels with age, most marked in the posterior circulation (2). In a follow-up study, aerobically active healthy elderly adults (68 ± 6 years) were demonstrated to have decreased vessel tortuosity and increased smaller vessels compared with less active age-matched subjects (3). Increased vascular tortuosity and arterial branch reductions in normal aging were also found in a study that evaluated 163 older adults (56–85 years), with associated age-related increased tortuosity mostly observed in middle cerebral artery/distal arteries (4). Previous MRA quantification techniques, however, have not been reported to reliably characterize infant vasculature due to the greater arterial and brain pulsation, and more diverse imaging artifacts at this early age (5, 6).

Intracranial artery feature extraction technique (iCafe) (1), a recently developed technique to quantify intracranial artery features, including length and tortuosity, has been validated with good to excellent reproducibly (1). With a recently reported artery refinement algorithm, iCafe has been shown also to reliably analyze MRA scans from challenging clinical populations, including infants (7).

In this paper, we applied the iCafe technique, along with our artery refinement algorithm (7), to study infants at two age points (7 months and 12 months) in comparison to a group of older adults. The goal of this study is to use the comprehensive and quantitative vascular features to explore vasculature developmental differences between infants and adults so that regional variations of blood supply can be assessed during early brain development and aging.

## Methods

### Study Population

No participants were scanned under sedation as all studies were non-clinical. 3D TOF MRA were acquired from 27 healthy infants (9.5 ± 2.4 month, eight females), comprised of 13 7-month-old infants (7.3 ± 1.0 month, four females) and 14 12-month-old infants (11.7 ± 0.4 month, four females), and 13 older healthy adults (62.8 ± 8.7 years, two females). Four infants were scanned at both 7-months and 12-months. No health problems, or medical conditions, were reported for any subjects at the time of scanning. These data were acquired as part of several MR imaging projects approved by the UW Institutional Review Board; written informed consent was obtained from the parents of all infants and from all adult participants before enrollment.

### MRA Data Acquisition

3D TOF MRA data were acquired on a 3.0 T Philips MR scanner (Ingenia CX, Best, The Netherlands) located at the University of Washington Biomolecular Imaging Center. For infant subjects, the following 3D-TOF MRA sequence parameters were used: TR 19.6 ms, TE 4.1 ms, flip-angle 18 degrees, axial plane, slice thickness 1.4 mm, interpolated resolution 0.35 × 0.35 × 0.35 mm, field of view (FOV) 150 × 150 mm, matrix size 216 × 214. For adult subjects, a 3D-TOF MRA sequence with the following parameters was used: TR 25 ms, TE 3.5 ms, flip-angle 20 degrees, axial plane slice thickness 1.4 mm, interpolated resolution 0.35 × 0.35 mm, FOV 180 × 180 mm, matrix size 370 × 278. The scan volume encompassed the cerebral vascular distribution with centerlines going through the anterior commissure-posterior commissure line and interhemispheric fissure on survey images. Infants were scanned while naturally asleep.

A subset of three adult subjects were rescanned using both adult and pediatric parameters to assess parameter effects on measurement quantification (See Supplemental Material).

### Feature Extraction

For all subjects, intracranial vasculature throughout the brain was traced using iCafe, a semi-automated artery tracing tool we have previously reported (8). iCafe is available for academic uses through our website (http://icafe.clatfd.cn).

In iCafe, arteries in TOF MRA were traced using an open-curve active contour model (9) and reconstructed as radius-varying tubes. To ease the artery tracing problems caused by artery boundary blurring due to pulsation and global image quality deterioration due to the motion during MRA scan, an artery trace refinement algorithm was used to correct centerline deviations and erroneous radius estimations along the traces. Then arteries were labeled as one of the 24 anatomical types (8, 10) so that comprehensive regional-based arterial features could be extracted, such as distal artery length and tortuosity of middle cerebral arteries. Three major vascular regions: anterior cerebral artery (ACA), middle cerebral artery (MCA), and posterior cerebral artery (PCA) were used for regional vascular feature analysis by grouping corresponding arteries from 24 anatomical types.

In addition to the features introduced in iCafe (1), several new features are introduced in this study.

To normalize the various brain sizes between subjects, the maximum brain area in axial dimensions was manually identified in each subject, and the brain region was selected using the “wand tool” with tolerance of 40 intensity difference provided in ImageJ (11). The number of pixels in the brain region was then defined as the maximal brain area. To evaluate density of arteries in specific brain regions (e.g., left or right M2+ segments for middle cerebral artery region), the minimum rectangular box that encompassed all arteries in the region was superimposed, then arterial density was defined as the volume of arteries in the region divided by the volume of the encompassing box.

**Figure 1:**
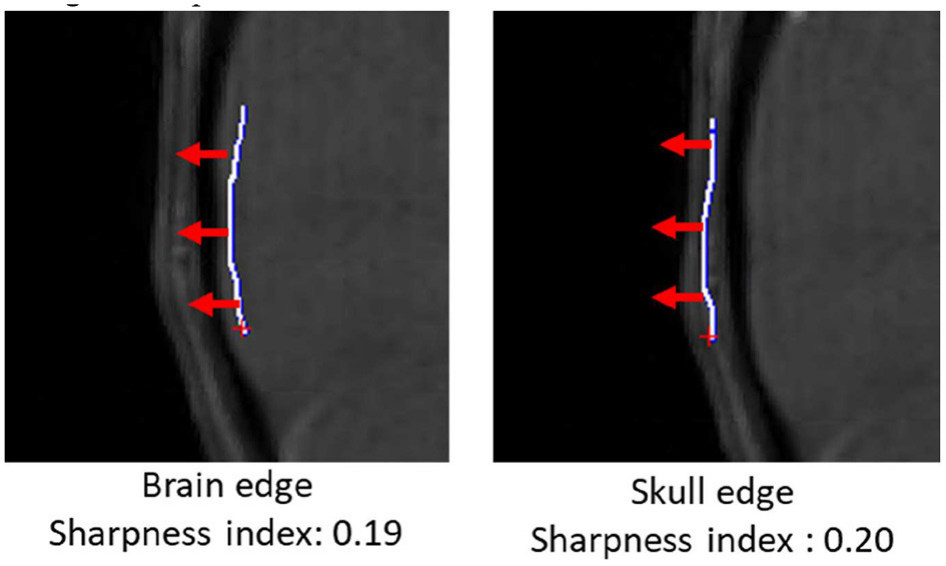
Brain (left) and skull (right) edges (white lines) drawn manually on an infant scan in iCafe for brain and skull sharpness index calculation. Gradient direction in red arrows (length of 10 pixels).

In addition to vascular morphometric features, brain and skull motion were separately quantified in order to evaluate effects of motion caused by vascular pulsation and gross motion caused by head movement. Samples of brain and skull edges were manually drawn in iCafe (Figure 1) so that intensity along the edges could be extracted. The maximum (within a range of 10 pixels) descending horizontal gradients outward from the brain center along the points on the edges were then identified. We defined a “sharpness index” as the mean of gradient divided by the mean intensity along the edges. Sharper edges have higher sharpness indexes.

### Statistical Analysis

The comprehensive morphometry and intensity features were compared between each age group using analysis of variance (ANOVA), with *post-hoc* comparisons using Student's *t* test. A two-tailed *p*-value of 0.05 was considered significant in consideration of the heuristic nature of this study.

### Vascular Measurements

The vascular features analyzed in this study included: total length and volume of artery branches [except the internal carotid artery (ICA), basilar artery (BA) and vertebral artery (VA) due to partial coverage by MRA); normalized (with maximum brain area) measures of length and volume, tortuosity, and density of M2+ area are additionally reported. Length measurements were further separated into ACA, MCA, and PCA regions to assess regional vascular length differences. We additionally compared left and right arterial features to assess for possible laterality differences.

Brain and skull sharpness indexes, and adult compared to pediatric acquisition parameters, were further assessed (included in Supplementary Material) to account for possible effects on reported measures.

## Results

### Infant and Adult Differences

**Figure 2:**
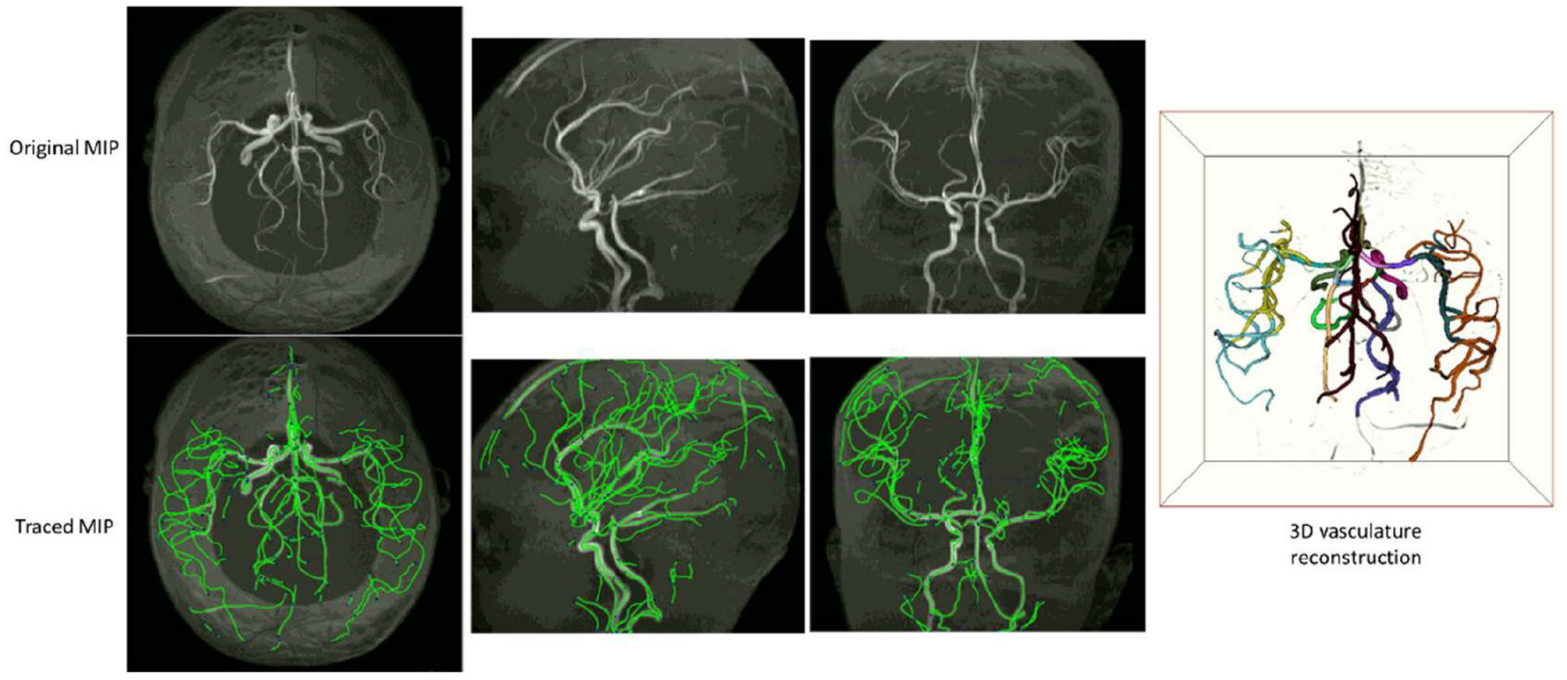
An example of iCafe processed infant in axial, sagittal, and coronal maximum intensity projection (MIP).

**Figure 3:**
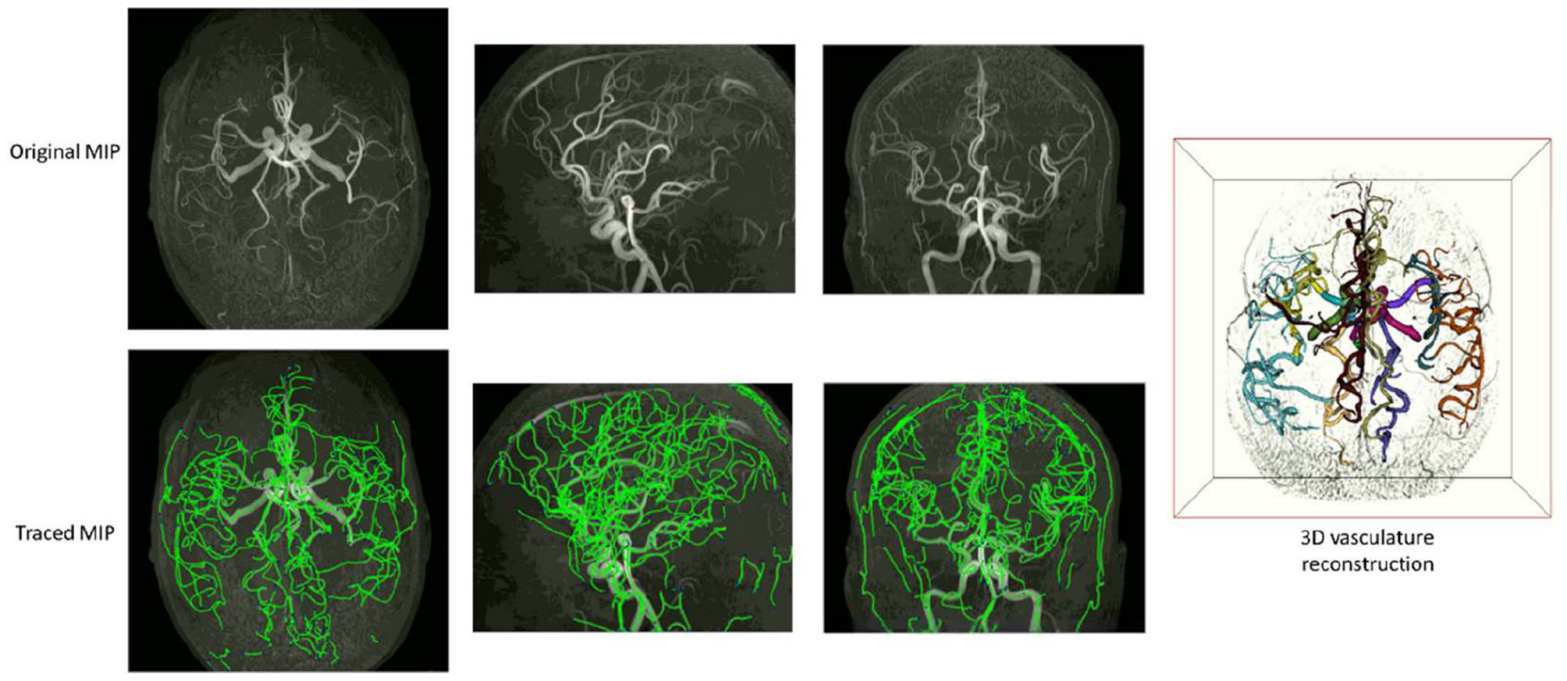
An example of iCafe processed adult in axial, sagittal, and coronal maximum intensity projection (MIP).

Examples of iCafe processed artery traces for infants and adults are shown in Figures 2, 3.

The artery feature differences between infants and adults are shown in Table 1. The infants, compared with adults, show significantly shorter artery length and smaller tortuosity, but higher volume and artery density. Skull and brain sharpness are reduced in infants.

**Table 1:** Infant and adults artery feature differences.

	**7-month infant mean**	**12-month infant mean**	**Adult mean**	**ANOVA *p*-value**	***T*** **test** ***p*****-value**		
					**7-month vs. 12-month**	**7-month vs. adults**	**12-month vs. adults**
Total length (mm)	2893.1	3180.4	3800.0	0.002	0.210	<0.001	0.015
Norm total length (mm)	3157.7	3193.7	3776.9	0.007	0.862	0.007	0.008
Total volume (mm^3^)	6732.3	8525.2	6714.8	0.060	0.079	0.978	0.050
Norm total volume (mm^3^)	7698.4	8574.9	6689.1	0.094	0.376	0.142	0.036
MCA length (mm)	1625.7	1798.7	2019.5	0.047	0.233	0.017	0.178
ACA length (mm)	746.5	855.3	1254.2	<0.001	0.085	<0.001	<0.001
PCA length (mm)	487.5	488.4	479.8	0.985	0.985	0.896	0.871
M2L+ Density 10e-3	7.47	9.77	5.38	<0.001	0.071	0.018	<0.001
M2R+ Density 10e-3	7.91	9.28	6.10	0.017	0.256	0.014	0.013
Number of branches	115.9	120.3	123.8	0.720	0.627	0.439	0.727
Average tortuosity	1.47	1.53	1.77	<0.001	0.020	<0.001	<0.001

The brain/skull sharpness indexes are 0.15/0.16, 0.15/0.17, and 0.18/0.19 for 7-month infant, 12-month infant, and adult. Infant groups show significantly smaller sharpness indexes compared with adults (*p*-values for *t* tests are all <0.001).

The vascular differences were not driven by brain or skull motions indicated by significant differences after controlling for brain or skull sharpness index in partial Pearson correlation (see Supplementary Material).

**Figure 4:**
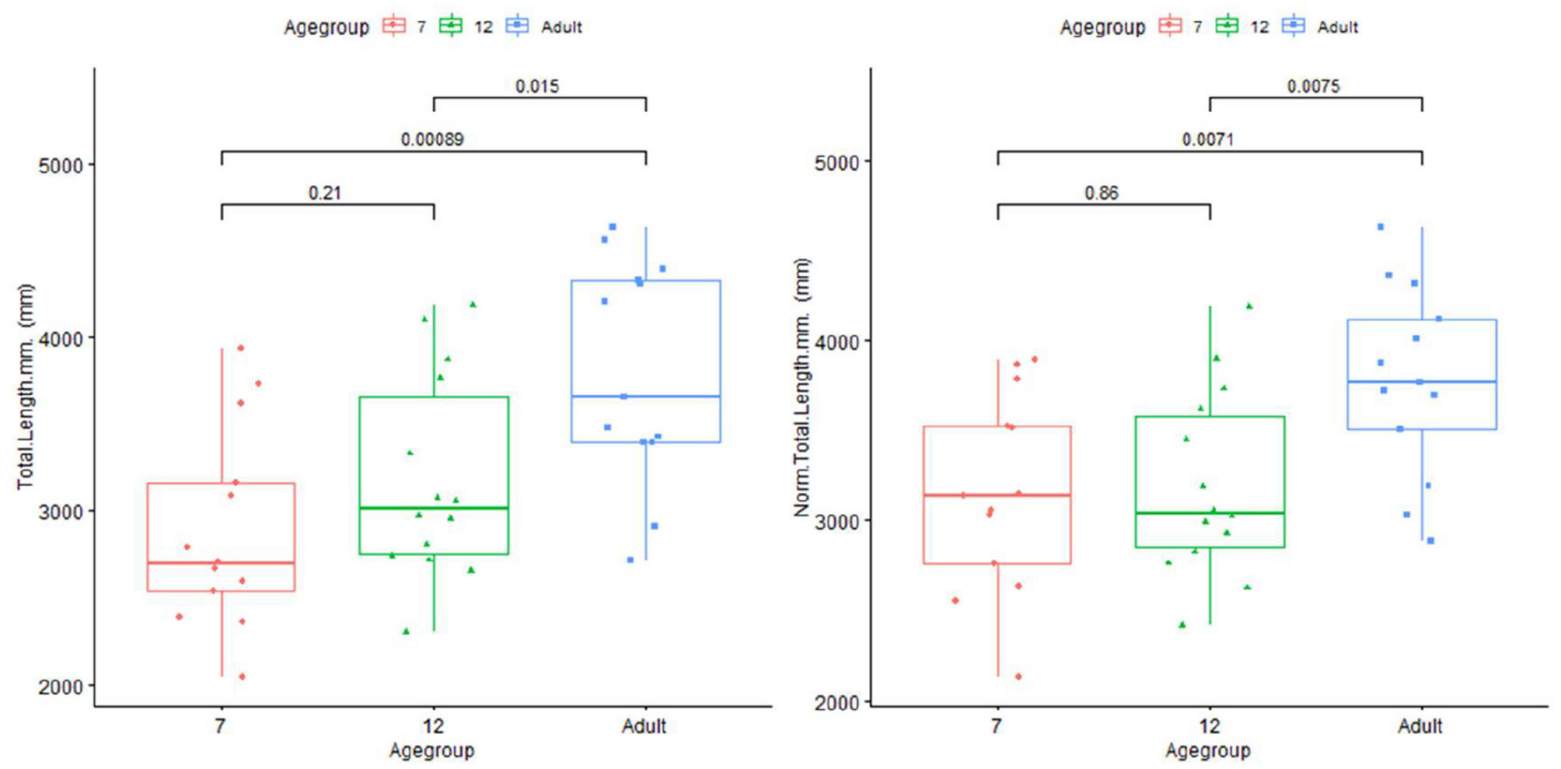
Plot for length and normalized length in infants (age group in months) and adults.

**Figure 5:**
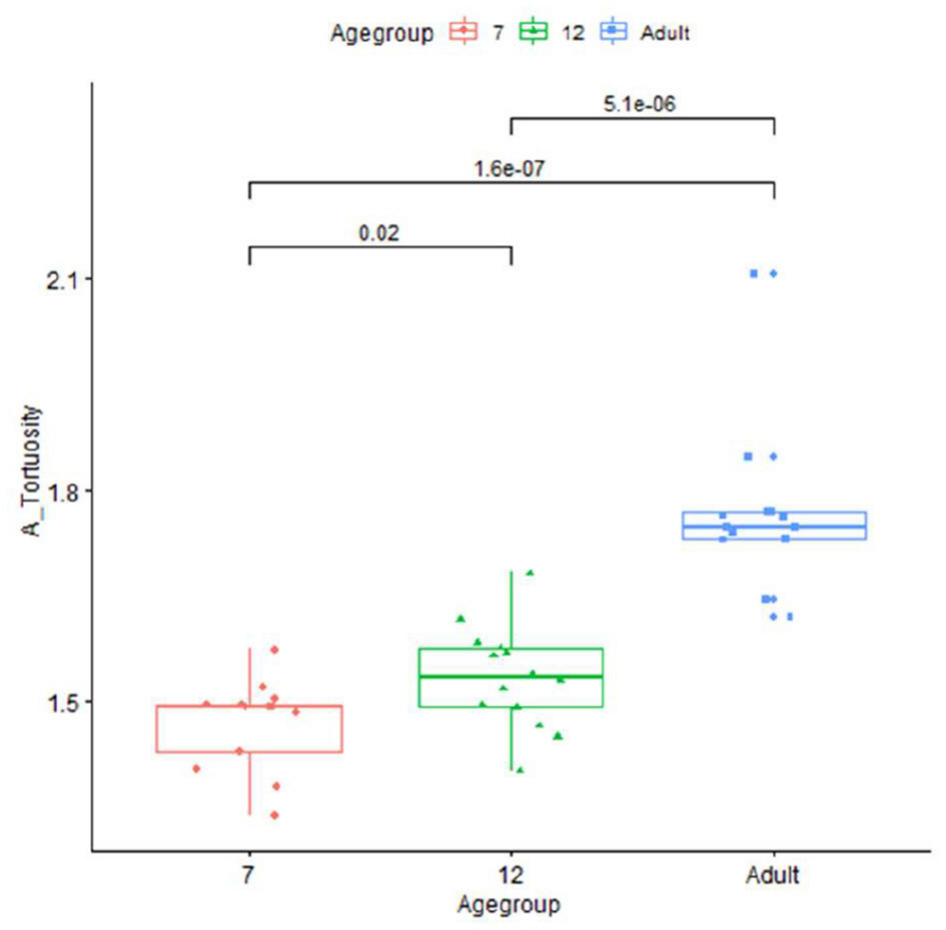
Plot for tortuosity in infants (age group in months) and adults.

**Figure 6:**
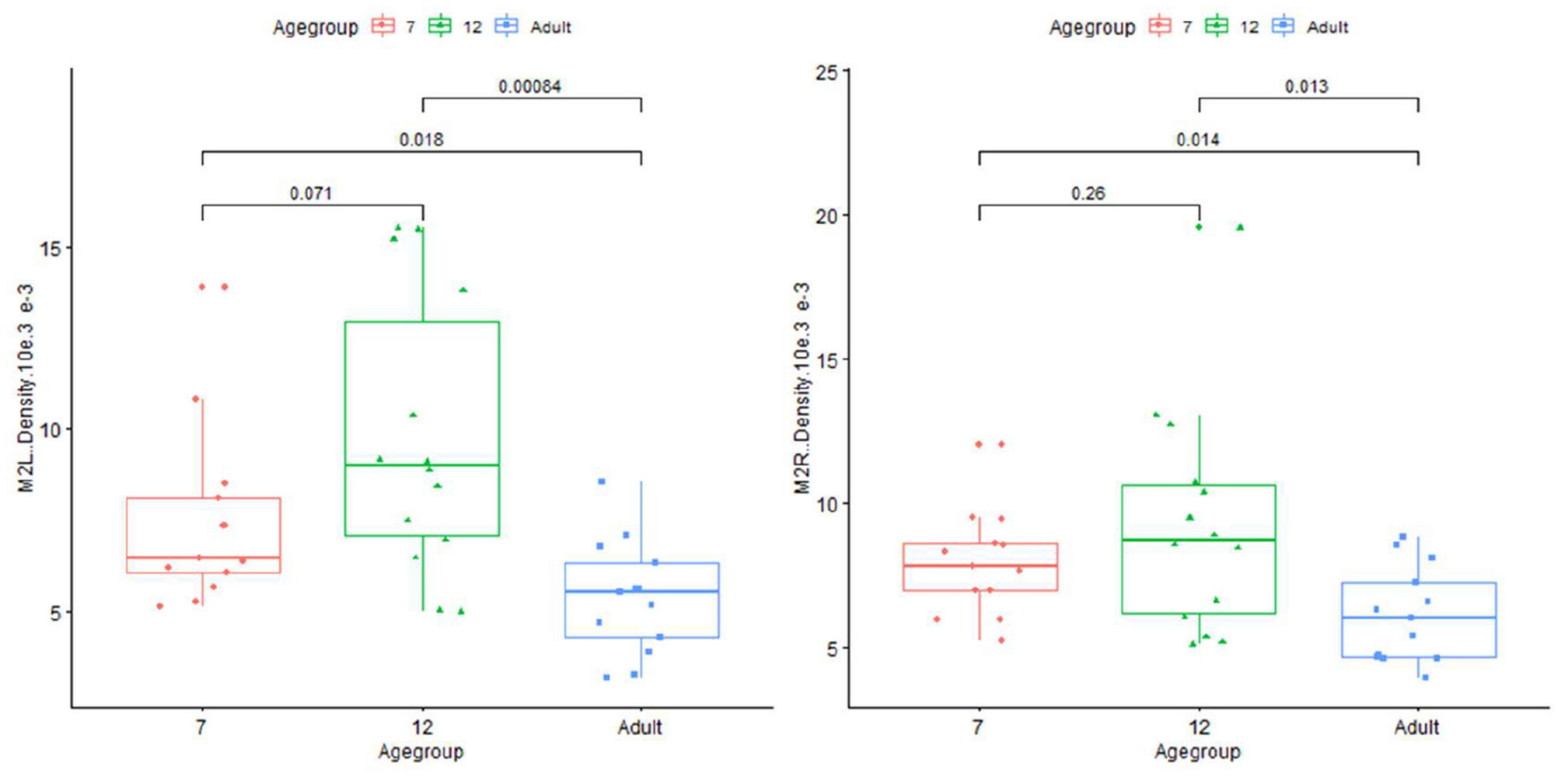
Plot for density on infant (age group in months) and adults in left and right M2+ segments of MCA.

**Figure 7:**
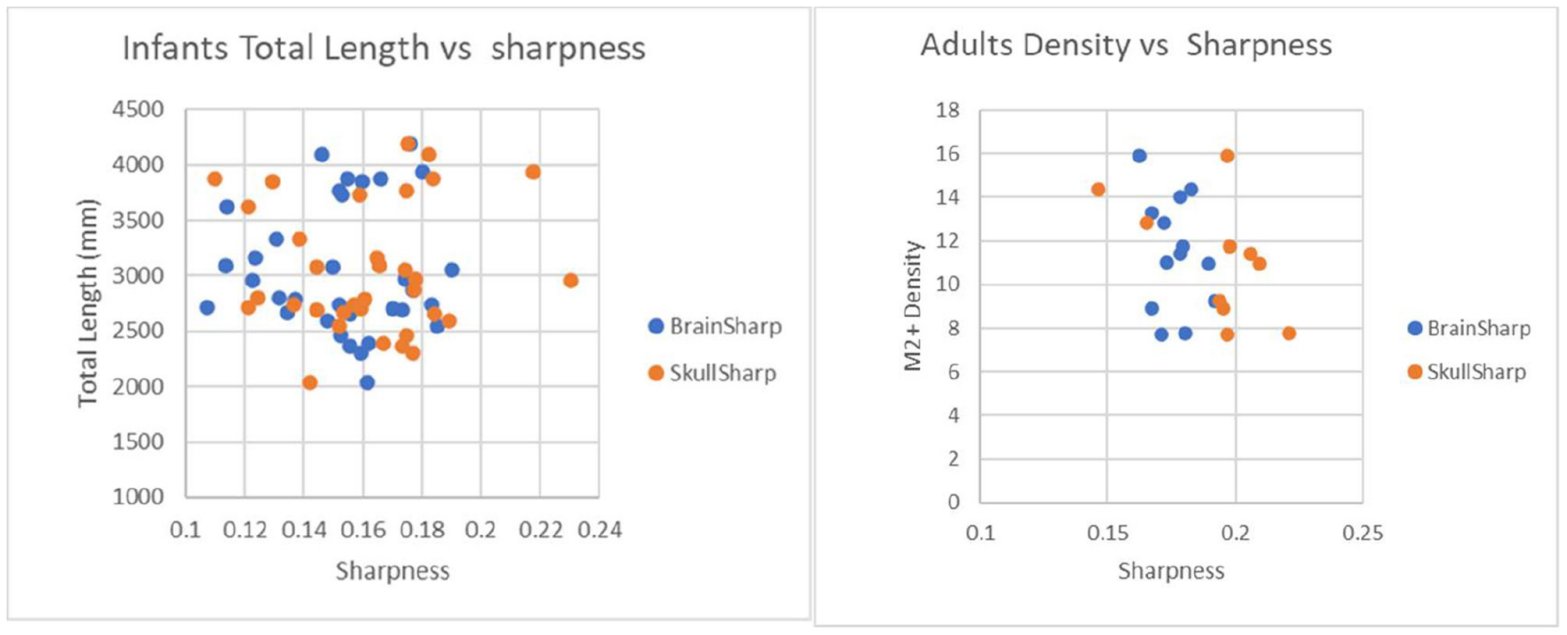
Plot to illustrate effects of brain sharpness and skull sharpness indexes on infant and adult vascular length measurements. *P*-values for correlations between brain/skull sharpness indexes and total length are 0.97/0.84 for infants and 0.31/0.61 for adults.

Plots for each infant group and adults on length, normalized length, tortuosity, density, and sharpness are shown in Figures 4–7.

### Differences Between Vascular Regions

**Figure 8:**
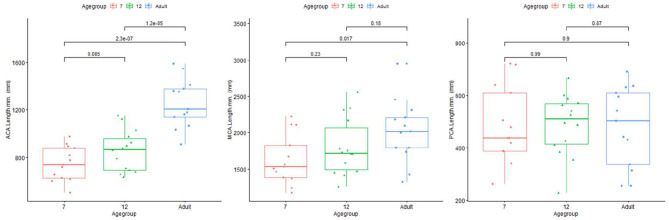
Plot for length of ACA (left), MCA (middle), and PCA (right) regions of infants and adults.

Length difference in MCA/ACA/PCA are shown in Figure 8. ACA length was found to be significantly longer in the adult group, with trend enlargement observed when comparing 12-month to 7-month-old infants. MCA length showed trend overall age differences, with significantly increased length observed in adults compared to 7-month-old infants. PCA length did not demonstrate age relationships.

### Laterality Differences

The number of branches, artery length, and volume were compared for left vs. right differences, shown in Table 2. No significant laterality differences in either infants or adults were identified.

**Table 2:** Left and right sided number of branches, length and volume in infants and adults.

	**7 Month**			**12 Month**			**Adults**		
	**Branch**	**Length**	**Volume**	**Branch**	**Length**	**Volume**	**Branch**	**Length**	**Volume**
Mean left	60.1	1475.3	3325.9	58.0	1530.7	4065.1	64.2	1938.9	5222.8
Mean right	55.2	1415.1	3392.9	61.4	1644.4	4435.6	59.2	1865.6	4725.2
*t* test *p*-value	0.122	0.334	0.732	0.349	0.219	0.242	0.162	0.37	0.363

## Discussion

Comprehensive intracranial artery analysis of MRA acquisitions was performed on potentially challenging datasets of non-sedated infants in comparison to older adults using the technique of iCafe, combined with an artery refinement algorithm developed for pediatric MRA due to potential limitations of increased skull movement during natural sleep and greater brain/vascular pulsation (7). This approach, along with normalization of assessed brain area, provides systematic and objective assessment of the arterial features extractable from the TOF MRA used clinically in non-invasive imaging of human intracranial arteries across the whole human life spectrum. Using both morphometric and intensity features extracted from the pediatric MRA, we were able to quantitatively analyze arterial characteristics of the infant groups and compare these features with those in older adults.

This study is an important contribution to the existing work on quantitative intracranial artery feature analysis. Instead of analyzing only adults (age 18+) (2, 12, 13), we extended the age range to infants. More importantly, compared with previous work, which provided only limited measurements such as artery length and tortuosity (4, 14–16), we developed new metrics to evaluate artery density and brain/skull sharpness, which extended our analysis capabilities evaluating intracranial MRA, particularly in pediatric populations.

Age-related arterial feature differences observed in this study are explainable and may contribute to a greater understanding of human cerebral vasculature developmental growth and disease development. Observations of age-related increases in arterial length in the older adults would be consistent with continuing arterial growth beyond early childhood. PET studies demonstrate dramatic, rapidly increasing brain metabolic activity during early development (17), then progressive declines with aging that is postulated to reflect an initial process of overproduction, and then subsequent elimination of excessive neurons, synapses, and dendritic spines (18, 19). Those considerations would be consistent with our observations of a trend increase in vascular density between 7 and 12 months that then is significantly decreased bilaterally in older adulthood. In addition, the systemic analysis of intracranial arteries by iCafe could have utilities in clinical studies of vascular disease states, such as Moyamoya (20) or mutations in the skeletal muscle α-actin gene (ACTA2) in children and cardiovascular disease or strokes in adults (21, 22).

Our vascular quantification approach was able to robustly detect individual variations, for example, substantial variation in infant length measurements, that may have research and clinical utility. Differences in length between MCA, PCA, and ACA also suggest that regional arterial growth is not uniform during development. Fewer length changes were observed in the PCA, which may reflect a differential lobar growth pattern. The much greater tortuosity measures in adults, consistent with what is observed clinically, may be related to the occurrence of age-related brain atrophy, as previously suggested in the literature (23, 24). Alternatively, increased vessel tortuosity could reflect endothelial turnover where there is actual vessel lengthening that occurs with aging, without increase in the intracranial volume (25). The vascular measurements were essentially unchanged, comparing MRA acquired in a subset of adults using both adult and pediatric acquisition parameters, which supports that iCafe is measuring actual age-related differences in vascular features, rather than reflecting different acquisition parameters.

Skull sharpness index is more related to gross head movement during scanning, while the brain sharpness index is affected by both head motion and pulsation related to blood flow. By quantitatively extracting the sharpness features, we can quantify and observe the relative impact of head motion in infants. A lower brain sharpness index in infants is expected because the infants have much higher pulse rate and hyperdynamic vascular response. The skull sharpness index is greater than the brain sharpness index in infants since, in addition to vascular pulsation, the brain edge is also affected by the same gross motion as skull.

In adults, the value of automated quantitative vascular imaging measures to investigate central nervous system diseases has been demonstrated (4, 16). Though less commonly applied in the pediatric clinical setting, the utility of systematic, quantitative vascular imaging evaluation has been demonstrated in recent work investigating childhood arteriopathy that found increased tortuosity measures in transient cerebral arteriopathies (TCA) and arterial dissection compared to controls, with the authors speculating that quantified arterial tortuosity could represent a relevant imaging biomarker (26, 27). In those studies, the quantitative approach utilized was limited to artery tortuosity measurements of major arterial branches without reproducibility assessments. In contrast, the iCafe approach allows investigation of additional morphologic features, such as artery length and density (1, 4, 10).

Clinical entities with cerebral vasculature pathology can first emerge in childhood, such as Moyamoya disease or genetic defects such as Neurofibromatosis type 1 (NF-1), where progressive narrowing of central cerebral vasculature results in progressive ischemic brain injury. This progressive vasculopathy can overlap in appearance with a non-progressive form of vasculopathy, categorized as focal cerebral arteriopathy (FCA). FCA occurs within the larger umbrella of TCA, which also includes similar appearing arteriopathies with known viral association, such as post-Varicella infection (28). Distinguishing the typically self-limiting TCA from the progressive arteriopathy of Moyamoya or NF-1 genetic defects can be problematic, acutely at times, with substantial treatment implications as to which diagnosis is given. Systematic vascular evaluation, such as with using iCafe, may have value for further understanding and, ultimately, clinical management of these pediatric vascular entities. In a different vein, identification of mutations in α-actin gene [ACTA2], which results in narrowing of cerebral vasculature with marked diminishment in length and normal tortuosity, presents another potential clinical setting for the systematic evaluation of pediatric cerebral vasculature. The discovery of such genetic variation in vessel morphology raises the prospect of employing systematic evaluation of cerebral arteries correlated with genetic data to further elucidate possible mechanisms in cerebrovascular disease.

There are several limitations to this study. As a preliminary study, this is a primarily cross-sectional investigation of a relatively small number of infants and adults, which aims to apply artery quantification techniques recently developed for pediatric subjects and explore potential developmental differences between infants and adults. Longitudinal assessment of a larger pediatric cohort will be useful in the future to further validate and extend our findings in this study. Including older children will also be of value; however, likely much of the informative feature changes will occur within the first 2 years of life (17), when maximal synaptic density and at least 80% of brain growth occurs.

The impact of inconsistent image quality among infants was generally addressed by an artery refinement algorithm specifically developed for this purpose (10), but it cannot be entirely eliminated, which might cause some bias in quantifying vascular features. Additionally, the iCafe process is semi-automated, and there are manual trace editing and manual brain area measurement steps for acquiring the features, which limits the number of cases that can be rapidly analyzed, and might also introduce operator-generated noise in quantitative vascular features.

In this study, iCafe was used to quantify morphometry and intensity features of intracranial arteries in infants and older adults. Systematic quantification of cerebral vasculature, such as the features obtained in this study, could help to understand aspects of brain vascular development and aging. As well, this type of systematic analysis may have clinical utility for assessing and managing pediatric and adult vascular diseases.

## Data Availability Statement

The data analyzed in this study is subject to the following licenses/restrictions: Subject data cannot be shared publicly. Requests to access these datasets should be directed to Chun Yuan, cyuan@uw.edu.

## Ethics Statement

The studies involving human participants were reviewed and approved by UW Institutional Review Board. Written informed consent to participate in this study was provided by the participants' legal guardian/next of kin.

## Author Contributions

All authors listed have made a substantial, direct and intellectual contribution to the work, and approved it for publication.

## Conflict of Interest

The authors declare that the research was conducted in the absence of any commercial or financial relationships that could be construed as a potential conflict of interest.

## Abbreviations

iCafe: IntraCranial artery feature extraction
ACA: Anterior cerebral artery
MCA: Middle cerebral artery
PCA: Posterior cerebral artery
BA: Basilar artery
VA: Vertebral artery
TCA: Transient Cerebral Arteriopathies
FCA: Focal cerebral arteriopathy.
