# Supplementary material for: Quantitative Assessment of the Intracranial Vasculature of Infants and Adults Using iCafe (Intracranial Artery Feature Extraction)
Source: Front Neurol. 2021 May 28;12:668298. doi: 10.3389/fneur.2021.668298 (PMC8193571; doi:10.3389/fneur.2021.668298)
Supplement: Supplementary file 1 [file Table_1.DOCX]

Supplementary material

Brain and skull sharpness indexes were used as the controlling variables to test whether vascular features are still different between age groups. Results are shown in Table 1, indicating brain and skull sharpness indexes are not the driving factor for the vascular differences.

TABLE 1 p value for t test between Infant and adults on artery feature differences controlling for brain and skull sharpness index

|  | 7-month vs 12-month | | 7-month vs Adults | | 12-month vs Adults | |
| --- | --- | --- | --- | --- | --- | --- |
|  | Controlling for Brain Sharp | Controlling for Skull Sharpness | Controlling for Brain Sharp | Controlling for Skull Sharpness | Controlling for Brain Sharp | Controlling for Skull Sharpness |
| Total Length(mm) | 0.222 | 0.242 | 0.005 | 0.019 | 0.182 | 0.072 |
| Norm Total Length(mm) | 0.844 | 0.889 | 0.011 | 0.049 | 0.083 | 0.018 |
| Total Volume(mm^3) | 0.084 | 0.082 | 0.429 | 0.672 | 0.058 | 0.085 |
| Norm Total Volume(mm^3) | 0.358 | 0.337 | 0.919 | 0.645 | 0.090 | 0.136 |
| MCA Length(mm) | 0.250 | 0.271 | 0.041 | 0.089 | 0.705 | 0.435 |
| ACA Length(mm) | 0.093 | 0.101 | <0.001 | <0.001 | 0.001 | <0.001 |
| PCA Length(mm) | 0.985 | 0.952 | 0.994 | 0.551 | 0.985 | 0.578 |
| M2L+ Density 10e-3 | 0.057 | 0.040 | 0.611 | 0.205 | 0.025 | 0.013 |
| M2R+ Density 10e-3 | 0.272 | 0.236 | 0.210 | 0.573 | 0.023 | 0.061 |
| Number of Branches | 0.641 | 0.699 | 0.500 | 0.960 | 0.943 | 0.989 |
| Average Tortuosity | 0.018 | 0.022 | <0.001 | <0.001 | <0.001 | <0.001 |
| Brain Sharp Index | N/A | 0.944 | N/A | 0.024 | N/A | 0.010 |
| Skull Sharp Index | 0.732 | N/A | 0.062 | N/A | 0.175 | N/A |

Three adults were scanned using both pediatric and adult acquisition parameters to evaluate the impact of imaging method on vascular quantification. All of the features except the brain sharpness index showed significant differences (results in Table 2), indicating the quantification method is robust to both acquisition parameters.

TABLE 2 Adults having both protocol

|  | Adult protocol | Infant protocol | t test p value |
| --- | --- | --- | --- |
| Total Length(mm) | 3190.0 | 3270.6 | 0.744 |
| Norm Total Length(mm) | 1116.1 | 1165.7 | 0.700 |
| Total Volume(mm^3) | 10605.0 | 8704.0 | 0.315 |
| Norm Total Volume(mm^3) | 448.2 | 394.0 | 0.505 |
| MCA Length(mm) | 1729.7 | 1771.4 | 0.857 |
| ACA Length(mm) | 1054.1 | 1084.5 | 0.803 |
| PCA Length(mm) | 354.8 | 356.5 | 0.983 |
| M2L+ Density 10e-3 | 9.15 | 7.30 | 0.549 |
| M2R+ Density 10e-3 | 10.48 | 6.97 | 0.262 |
| Number of Branches | 104.3 | 108.3 | 0.687 |
| Average Tortuosity | 1.88 | 1.80 | 0.547 |
| Brain Sharpness Index | 0.16 | 0.12 | 0.008 |
| Skull Sharpness Index | 0.20 | 0.15 | 0.267 |
